# Generation COVID-19 and bodily disorders: Hyperbolic narratives and a developmental psychopathology perspective

**DOI:** 10.3389/fpubh.2022.976584

**Published:** 2022-08-12

**Authors:** Alessandra D'Agostino, Emanuela Saveria Gritti, Chiara Gagliardi

**Affiliations:** ^1^Department of Humanities, University of Urbino Carlo Bo, Urbino, Italy; ^2^Department of Psychology, University of Milano-Bicocca, Milan, Italy

**Keywords:** bodily disorders, non-suicidal self-injury, eating disorders, adolescents, COVID-19, developmental psychopathology

## Abstract

Starting from spring 2020, newspapers headlines and studies have suggested that the COVID-19 pandemics had a negative impact especially on the mental health of children and adolescents, so that terms like “lost generation” or “generation COVID-19” have been used to define youth in time of pandemic crisis. Similarly, international health agencies reported an increase in depression and anxiety among adolescents in COVID-19 time, but also a rise in bodily disorders, such as non-suicidal self-injury and eating disorders. However, scientific data on this matter are not as clear as they seem and theoretical-clinical proposals regarding the processes involved are lacking. Focusing specifically on bodily disorders in adolescents during COVID-19, the aim of this perspective paper is to review this issue and propose a novel viewpoint on it. Firstly, data regarding frequency and phenomenology of bodily disorders in adolescence before and during the pandemic will be presented to underline possible discrepancies, gaps, or hyperbolic descriptions in the literature published after the COVID-19 outbreak. Secondly, a specific theoretical-clinical perspective will be proposed, that is, a developmental psychopathology perspective which attempts to frame these phenomena in a more nuanced and complex way, taking into account the role of developmental processes in adolescence age and its difficulties in the specific, subjective life-context of the individual, when intertwining with vulnerability factors and stressful life events. As such, the function of the body for the adolescent as a primary mean for regulating the self-other relationship and developing a greater sense of self-agency will be highlighted. The final objective is to help the clinician in developing both a critical thinking about the data that are shared in public outlets and an intervention that takes into account the complexity of contemporary psychopathological phenomena.

## Introduction

Since the beginning of the COVID-19 pandemic in 2020, media and journals have highlighted an increasingly serious alarm regarding its negative effects on the mental health of young people, so that terms like “lost generation” or “generation COVID-19” have been used to identify youth in time of pandemic crisis, that is, those who have had points of transition in their life disrupted (1–5).

Similarly, WHO (6) and other international health agencies (7), together with a certain number of studies (8–14) reported an increase in depression and anxiety among adolescents in COVID-19 time, but also a rise in bodily disorders, such as non-suicidal self-injury and eating disorders. These data, if absolutized, shed a dark light on the future of mental health of youth. However, things are not always as simple as they seem, especially when psychopathology is at stake.

Focusing specifically on bodily disorders in adolescents during COVID-19, the aim of this perspective paper is to review this issue and propose a novel viewpoint on it. Firstly, data regarding frequency and phenomenology of bodily disorders in adolescence before and during the pandemic will be presented to underline possible discrepancies, gaps, or hyperbolic descriptions in the literature published after the COVID-19 outbreak. Secondly, a specific theoretical-clinical perspective will be proposed, that is, a developmental psychopathology perspective which attempts to frame these phenomena in a more nuanced and complex way, taking into account the role of developmental processes in adolescence age and its difficulties in the specific, subjective life-context of the individual, when intertwining with vulnerability factors and stressful life events. As such, the function of the body for the adolescent as a primary mean for regulating the self-other relationship and developing a greater sense of self-agency will be highlighted.

The final objective is to help the clinician in developing both a critical thinking about the data that are shared in public outlet and an intervention that takes into account the complexity of contemporary psychopathological phenomena.

## Bodily disorders in adolescents during pandemic time: What do we know so far?

From a quickly glance at the media and newspapers headlines dated 2020–22 (15–17), and especially the Italian ones (18–23), the impression is of a real explosion of psychopathological phenomena related to the body in adolescents, since the COVID-19 pandemic has begun. In particular, non-suicidal self-injury (NSSI) (24), i.e., deliberately injuring one's own body tissue without suicidal intent, such as cutting or burning, and eating disorders (EDs), i.e., disturbed eating-related behaviors, such as anorexia or bulimia nervosa, appear to have increased dramatically in the young population over the past 2 years. But is the current situation really explosive or is there a hyperbole in its narration? To clarify things, it is necessary to look at the data provided by recent scientific literature. The main findings can be divided into three topics: (a) epidemiological data; (b) risk factors; (c) access to healthcare.

*First of all, epidemiological data*. Regarding NSSI, as underlined by Plener (25), there is currently a lack of knowledge of the impact of COVID-19 specifically on the prevalence of NSSI in adolescents, as most of the studies do not clearly distinct suicidal from non-suicidal behaviors. The few existing ones across different countries find an increase during the pandemic compared to pre-pandemic time, with cutting as the most prevalent method. For example, Zetterqvist et al. (26), analyzing life-time prevalence of NSSI in high school students in Sweden at three different time points, report very similar percentages of NSSI in 2011 and 2014 (17.2 vs. 17.7%), and an increase to 27.6% during the 2020–2021 period; however, authors specify that it is not clear if that increase is due to the pandemic or it appeared already before its onset. Similarly, Tang et al. (27) highlight a NSSI prevalence of 40.9% among 1.060 junior high school students in Taiwan during the COVID-19 outbreak (cutting = 21.6%), a rate estimated as increasingly higher than that found in previous international investigations on adolescent samples (rates between 13 and 36%). However, they also specify that no causal relationships could be determined due to the cross-sectional nature of the study. Interestingly, in most of the studies the prevalence of NSSI is significantly higher in girls and transgender/non-binary adolescents rather than in boys, and this seems valid in both pre- and post-pandemic times.

Regarding EDs, some studies report an increase in the number and severity of new and pre-existing adolescents suffering with eating disorders during the pandemic compared to pre-pandemic time, particularly for anorexia nervosa. For example, Taquet et al. (28), analyzing the electronic health records (EHR) of 5.2 million US people aged under 30 (mean age = 15.4 years), find that, after a decrease in the early portion of 2020 (which could reflect the marked reduction in all diagnoses made in EHR network during that period), the incidence of a first diagnosis of an ED has increased of 15.3% throughout the rest of 2020 compared to previous years, such that the relative risk had exceeded 1.5 by the end of the year. However, authors specify that the increase was limited to girls and mostly related to anorexia nervosa diagnoses. Moreover, Gao et al. (29), in a systematic review of recent studies on ED patients and COVID-19, observe that women and young people had greater concern about their body image and appearance, more difficulties in regulating eating, and a greater risk of worsening ED symptoms during the lockdowns. Conversely, authors also underline that some participants with anorexia nervosa reported relieving symptoms during the same period, probably thanks to e-therapy, more stable family relationships, and fewer social stressors. Finally, Graell et al. (30), conducting a study on Spanish young people with EDs, find that the patients with most severe psychopathology (25%) presented both NSSI and reactivation of ED symptoms.

*Secondly, risk factors*. Regarding this matter, just a few studies so far have tried to identify the mediating/moderating effect of risk factors in the relationship between pandemic and emerging or worsening of NSSI or EDs. As for NSSI, Robillard et al. (13), for example, using a sample of Canadian adolescents, report that the relationship between COVID-19 pandemic stress and NSSI was fully mediated by two dimensions of emotion regulation (ER) difficulties, namely non-acceptance of emotional responses and limited access to ER strategies. Similarly, Tang et al. (27), using a sample of Taiwan teenagers, suggest that the NSSI group during the pandemic mostly consisted of girls scoring significantly higher in neuroticism, depression, impulsivity, alexithymia, virtual social support, dissatisfaction with academic performance, and lower in subjective wellbeing, self-esteem, actual social support, as well as family function than the non-NSSI group. As for EDs, Cooper et al. (31), for example, recommend to take into account two types of risk factors that may increase ED risk during and following the COVID-19 pandemic: (a) eating-disorder specific risk factors (i.e., food insecurity, fatphobic messaging, and restricted healthcare access); (b) broader risk factors (i.e., stressful life events, anxiety, social isolation and decreased social support, trauma and abuse, perfectionist expectations, and gender role stress). Similarly, Linardon et al. (32), in a systematic scoping review of research on COVID-19 impact on EDs, find that those most susceptible to symptoms escalation and mental health worsening during pandemic were: confirmed eating disorder patients, at-risk populations (young women, athletes, parent/carers), and individuals highly anxious or fearful of COVID-19.

*Thirdly, access to healthcare*. Regarding NSSI, findings from different countries report a significative reduction in the use of psychiatric services by children and young people during the early phase of the pandemic. For example, Ougrin et al. (33), in a retrospective cohort study examining the differences in hospital emergency psychiatric presentations for non-suicidal self-injury of children and adolescents during the COVID-19 lockdown in March-April 2020 compared with the same period in 2019, find a noticeable decrease in recorded emergency presentations and in inpatient psychiatric admissions during the COVID-19 lockdown. Similarly, Yunus et al. (34), in a systematic review analyzing studies published between January 2020 and March 2021, show a considerable reduction in the use of psychiatric services by children and young people aged 0–24 during the initial phase of the pandemic, compared to pre-pandemic time. According to Chen (35), this reduction could be merely due to the lockdown restrictions, but also to other reasons, such as fear of being infected by COVID-19 or reduction of psychosocial risk factors during lockdown, such as less academic pressure and peer bullying and increased parental care. However, as highlighted by Plener (25) and remarked by Zetterqvist (26), most of the studies on this topic do not clearly separate suicidal from non-suicidal behaviors, so any specifically inference on the latter is risky and incorrect.

Regarding EDs, available literature is lacking systematic reviews, whereas single published studies report an increase in the healthcare access by teens with eating disorders during the pandemic, compared to pre-pandemic time. For example, Otto et al. (36), collecting data on patients aged 10–23 years admitted to their children's hospital in Michigan for restrictive EDs from March 2017 through March 2021, find that medical admissions among adolescents with EDs increased significantly during the COVID-19 pandemic, with the number of admissions during the first 12 months of the pandemic more than double the mean for the previous 3 years. Similarly, Lin et al. (37), analyzing monthly summary data on ED admissions to young medical services in children's hospital in Boston from January 2018 to February 2021, highlight that inpatient admissions, hospital bed-days, and outpatient care-related inquiries increased on average over time post-pandemic compared to pre-pandemic time. However, outpatient assessments decreased precipitously initially following COVID-19-related limitations, and rose quickly back to baseline. This latter finding is in line with the reduction of access to healthcare services by young people presenting non-suicidal self-injury, reported by the aforementioned studies. Nevertheless, such studies do not allow data generalization, as they reflect the experience of a single institution in a specific geographic area having also specific COVID-19 conditions and restrictions, as the authors themselves specify in their final considerations.

## Beyond the pandemic: The role of the body for the adolescent and an integrative framework to understand

This quick and certainly not exhaustive overview of the most relevant data available on bodily disorders in adolescents during the COVID-19 pandemic highlights a far more complex and nuanced scenario than what appears from newspapers headlines.

*First of all, increase is not (sudden) explosion*. The fact that NSSI and EDs in young people may have increased during the pandemic does not mean that they are born in pandemic time. Rather, it means that such phenomena must be evaluated in a much broader time perspective, as they were already widespread in the young population long before the pandemic, and probably also underestimated due to the social stigma frequently related to these disorders, that might obstruct help-seeking behaviors and contribute to decreased visibility and poor general awareness of these disorders in society (35, 38). The scientific community has been signaling an alarm in this sense for at least 10 years (39, 40), but media attention has only recently risen significantly.

*Secondly, correlation is not causation*. The fact that bodily disorders in adolescents have potentially increased *during* the pandemic does not mean automatically that they have increased *because of* the pandemic. Various mediating/moderating factors could be involved in this relationship, but they have not been considered in the published studies. This issue brings out another closely related one: significant methodological limits in the clinical studies published during the COVID-19 pandemic years call for caution in interpreting findings (6, 41, 42). In fact, most of them were observational, involved convenient samples, underwent shorter peer-review evaluation, and made comparison to pre-pandemic experiences that were retrospective recall or comparison to pre-pandemic cohorts without adequate control of differences between samples.

*Thirdly, and most importantly, psychopathology is a complex matter* involving multiple factors to account for single phenomena. According to cited findings, not all adolescents have experienced bodily disorders during the pandemic, and those who encountered difficulties in this regard have done it in different ways. For example, at-risk subgroups of adolescents (i.e., adolescents with pre-existing mental difficulties) are identified in all studies as the most prone to bodily disorders during the lockdown, although some of them report relieving rather than worsening symptoms during the same period, thanks to better family conditions and fewer social stressors. So, understanding such behaviors is not so simple and there is need to open up the perspective. In other words, psychopathological conditions must be located in the complex intertwining of life-context and mind-body relationship of the individual. Reasons and meanings must also be explored.

For all these reasons and given the urgency of developing a tailored intervention for these contemporary conditions, we propose to place bodily disorders in adolescents during the pandemic within the theoretical-clinical framework of developmental psychopathology perspective. Developmental psychopathology is an interdisciplinary field that has been developed between late 1970 s and early 1980 s (43, 44) and can be defined as “an evolving scientific discipline whose predominant focus is elucidating the interplay among the biological, psychological, and social contextual aspects of normal and abnormal development across the life span” [(45), p.1]. This field is distinct from clinical child psychology and focuses on the origins and courses of individual patters of behavioral maladaptation, with a particular attention to both risk and protective factors that delineate pathways of risk and resilience (46), thus painting a more complicated, flexible picture of psychopathology emerging through the combination of many factors shifting across time, some of which are deeply rooted in our biology (i.e., genetic) and others exist in our outside environments (i.e., parenting). Today developmental psychopathology is considered “an integrative framework that links different scientific disciplines, theories, and research strategies to understand better how individuals adapt and develop risk for psychopathology” [(47), p.19].

This kind of integrative framework is especially useful for understanding psychopathology in youth (47). In fact, adolescence is a particularly compelling phase of development, carrying a heavy load of developmental tasks to perform, due to the intensity of the biological, psychological and social changes faced and calling for a profound reorganization of the identity. Adolescent is no longer a child but not yet an adult. “Consequently, the flux and renegotiation inherent in this developmental period increase the potential for both internal and external conflict” [(48), p.6]. For all these reasons, adolescence has been defined as a period of “storm and stress” (49), and the problems in adjustment presented by a few were generalized as normative experiences for all adolescents (50). Certainly, the storm and stress of adolescence is neither universal nor inevitable (48). Nevertheless, adolescence does generate more psychological turmoil than either childhood or adulthood (51), thus bringing considerable stressors and compromising health (52).

Not surprisingly, literature and research underline a rise in rates of psychopathology during this phase (47, 53). Particularly, externalizing (e.g., aggression and conduct disorders) and internalizing problems (i.e., depression and anxiety) are two empirically derived dimensional constructs that have been used frequently to operationalize adolescent problems (54, 55), and that may be just transient symptoms disappearing in adulthood or, rather, start during childhood and continue into adolescence and beyond (56). Also, new problems such as personality disorders and eating disorders appear for the first-time during adolescence (57, 58). So, adolescence is a period characterized by both continuity and change in psychopathology (47, 56).

The body of the adolescent is the catalyst of all this “continuity and change.” As suggested by Diem-Wille [(59), p.4], also referring to Freud (60): “There is no period—aside from the time in the womb—when the body alters as much as in puberty. Bodily changes are subject neither to a person's will nor their control, erupting and eliciting fiery emotions in the adolescent.” Especially during this period of fundamental physical change, the body is most intimately linked to the ego (59). As Freud [(61), p.253] underlines: “The ego is always a body ego.”

Since all dimensions from the relatively peaceful latency period are in flux, the adolescent now finds a kind of refuge in his body, even though it is also a source of insecurities and fears as it rapidly changes. If he/she suddenly knows nothing about himself /herself (values, desires, position in the world), at least he/she can control his/her own body, or, better, that is the illusion.

Following this perspective, the body can be seen as an instrument -the one closest to hand- for the adolescent to adapt to the many internal/external changes, and its possible symptoms as a way for him/her to regulate the relationship between self and the world in a very turbulent time of life. This could be particularly true and violent in young people experiencing more severe psychopathology, for example those with comorbid internalizing and externalizing problems, who resemble a severe at-risk state in which the predictive value for psychopathology increases over time (56). In fact, in the developmental psychopathology view, disorders and diseases must be seen not as a static condition but as a dynamic transaction between intra and extra organismic forces, or as consequences of the active efforts of each individual to adapt to their environment (62). According to our perspective, bodily disorders (especially NSSI and EDs) can be located within this frame of meaning.

In fact, over the years several authors, clinicians, and scientists from different theoretical points of view have developed reflections and models to understand these behaviors. They all agree that bodily disorders have a specific function in the psychic economy of a person with a specific vulnerability in terms of body-mind integration and living specific environmental challenges. They are an immediately effective method, through the body, of regulating one's affective/cognitive experience and/or influencing one's social environment in a desired way, being maintained by intrapersonal or interpersonal vulnerability factors with reinforcement processes (63–72). In this view, bodily disorders in adolescents during the COVID-19 pandemic acquire a completely different depth compared to what appears from the newspapers: not incomprehensible “bolts from the blue,” but emerging phenomena within a complex dynamic of internal-external factors regulation in the subjective experience of a person in effort to relate to his/her life-context. We suggest to explore this whole network of risk and protective factors within a long-time span to understand complex clinical phenomena in present time and their possible course in the future.

## Discussion

A number of headlines referring to scientific studies have raised an alarm about a possible increase of bodily disorders, particularly NSSI and EDs, in adolescents during the COVID-19 pandemic. However, as highlighted in this paper, several issues call for caution in interpreting these data.

First of all, the true picture of mental health among young people is more complex than that portrayed in the news headlines (1). A hyperbolic style in recent news dramatically relating increased bodily disorders to the pandemic *per se* risks to obscure the complex network of factors influencing such relationship. The same can be said of studies highlighting the correlation between COVID-19 and increased disturbances without using the methodological complexity necessary to derive cause-and-effect relationships.

Secondly, the social stigma, often connected with this kind of disturbances, has not at all or little been considered in the present circumstances. As previously underlined, considerable stigma and self-stigmatization associated to NSSI and EDs might inhibit help-seeking behavior and contribute to poor general awareness of these disorders in society (38). In this sense, the pandemic might have exacerbated trends already present but not visible in the adolescent population, thus highlighting the status of adolescents' mental health (1) and legitimatizing somehow help-seeking behaviors (e.g., bodily disorders), previously held back by greater shame and fear.

Thirdly, risk and protective factors are imperative to map the course of these disorders and delineate psychopathological patterns that are dynamic and individual, rather than static and collective. So, conceptualizing such disturbances in a developmental psychopathology perspective can be helpful for bringing prevention and developing tailored treatments. It can also be useful for better identification of adolescents who are more prone to be resilient despite increasing current stressors, and those for whom, instead, increased exposure to stress could result in amplified psychological problems.

In conclusion, the pandemic has certainly had wide-ranging effects on young people, but we need to be more accurate to explore reasons and meanings in individual paths in order to understand questions concerning eventual psychopathology, as developmental plasticity and resilience typical of that stage of life give us room for optimism (5).

## Data availability statement

The original contributions presented in the study are included in the article, further inquiries can be directed to the corresponding author/s.

## Author contributions

AD conceptualized the work, designed the paper, and wrote the manuscript. ESG helped in conceptualizing the work, searching for literature and revising the manuscript. CG helped in revising the manuscript and preparing the submission. All authors contributed to the article and approved the submitted version.

## Conflict of interest

The authors declare that the research was conducted in the absence of any commercial or financial relationships that could be construed as a potential conflict of interest.

## Publisher's note

All claims expressed in this article are solely those of the authors and do not necessarily represent those of their affiliated organizations, or those of the publisher, the editors and the reviewers. Any product that may be evaluated in this article, or claim that may be made by its manufacturer, is not guaranteed or endorsed by the publisher.
